# Atrial fibrillation and alteration of heart rate variability after video-assisted pulmonary lobectomy versus thoracotomy pulmonary lobectomy

**DOI:** 10.1186/s13019-020-01260-6

**Published:** 2020-08-14

**Authors:** Gengxu He, Tong Yao, Lei Zhao, Hong Geng, Qiang Ji, Kun Zuo, Yuanzhi Luo

**Affiliations:** 1 Department of Thoracic and Cardiovascular Surgery, The First Affiliated Hospital of Hebei North University, Zhangjiakou City, Hebei Province P.R. China; 2 Department of Cardiac Function Examination, The First Affiliated Hospital of Hebei North University, Zhangjiakou City, Hebei Province P.R. China

**Keywords:** Atrial fibrillation, Video-assisted thoracic surgery, Thoracotomy, Heart rate variability

## Abstract

**Objective:**

To compare the incidence of atrial fibrillation (AF) and alteration of heart rate variability (HRV) after pulmonary lobectomy through video assisted thoracic surgery or thoracotomy, and to explore the role of autonomic nerves in the pathogenesis of atrial fibrillation after pulmonary lobectomy.

**Methods:**

In a single institution, 224 patients (age > 60) with normal sinus rhythm were enrolled in the study. Experienced surgeons and anesthetists carried out operation and anesthesia according to the same procedure. The hearts were monitored using Holter for more than 96 h. Any new-onset AF was recorded and HRV was analyzed at different time intervals.

**Results:**

One hundred twelve patients undergoing video-assisted thoracic surgery (VATS) and 112 patients undergoing thoracotomy (THOR) were matched for age and gender. Atrial fibrillation occurred in 39 patients, with a similar incidence between the two groups (VATS: 19/112, 16.9% and THOR: 20/112, 17.9%, *P* = 0.82). The post-operational heart variability at different time intervals was comparable between the two groups.

**Conclusion:**

Pulmonary lobectomy through video assisted thoracic surgery does not reduce the postoperative atrial fibrillation. Autonomic nerve mechanism may be involved in the pathogenesis of postoperative atrial fibrillation.

## Introduction

Atrial fibrillation (AF) is the most common arrhythmia after noncardiac thoracic surgery, with an incidence of > 20% in elderly people [1]. Postoperative AF contributes to increased morbidity and length of hospital stay, as well as greater risk of stroke (in those with persistent AF). The etiology of this complication remains poorly understood. Age of 60 years or older is consistently independent, preoperative risk factor for postoperative AF. Video assisted thoracic surgery (VATS) has been widely used for lobectomy attributed to non–rib spreading incision, a shorter hospital-stay and decreased acute postoperative pain [2]. Minimally invasive VATS technique may result in a higher rate of postoperative morbidity when compared with thoracotomy, especially in elderly or high-risk populations. Several studies reported that the incidence of AF could not be reduced with the use of VATS [3–5]. It was proposed that autonomic nerve play an important role in the pathogenesis of atrial fibrillation [6]. However, very few studies have been performed to compare individual incidence of AF and HRV between lobectomy through VATS and thoracotomy. Our study was designed to compare the incidence of AF between VATS and thoracotomy Alteration in postoperative heart variability at different time intervals has also been explored.

## Patients and methods

This study was approved by the institutional review board at the and informed consent was waivered. Prospectively selected patients were recruited into this study, with the inclusion criteria as follows: (1) clinical stage I lung cancer diagnosed by Computed Tomography, (2) age > 60, (3) sinus heart rhythm. Patients were excluded based on the following criteria: (1) not in sinus rhythm before surgery, (2) administered with class I or III antiarrhythmic drugs, or (3) treated with prophylactic medications postoperatively. Preoperative beta-blockers or calcium channel blockers for hypertension or coronary artery disease were re-administered on the first postoperative day to avoid withdrawal symptoms. Patients who underwent a lesser resection (exploration, wedge or segmentectomy) or more extensive operation (bilobectomy, pneumonectomy, chest wall resection, or major vascular resection) were excluded.

Standard anesthesia induction and maintenance regimens as well as intraoperative fluid restriction were applied for all patients. Postoperative pain relief was provided by continuous administration of epidural opioid (fentanyl with bupivacaine 0.05%). Anatomic pulmonary lobectomy and ipsilateral mediastinal lymph node dissection were performed by experienced thoracic surgeon to avoid bias in the procedure. The VATS lobectomy was defined as anatomic pulmonary lobectomy using a video thoracoscope and three non–rib spreading incisions. Patients converted from VATS for whatever reason were assigned to thoracotomy group. All patients were observed overnight in the post-anesthesia care unit on continuous telemetry and then transferred to a dedicated thoracic surgical ward on the first postoperative day.

Their hearts were monitored using Holter from 24 h preoperative to > 96 h postoperative. Dual-lead electrocardiography (leads CM2 and CM1 or CM5) was used to record on Marquette 8500 Holter. Recordings were made continuously upon arrival in the post-anesthesia care unit for 72 to 96 h. The Holter tapes were digitized on a Marquette series 8000 scanner. The signal was sampled at 128 Hz. The decision made automatically by the computer was reviewed and corrected by an experienced technician and then a cardiologist.

When calculating the HRV parameters, only normal-to-normal (NN) intervals were used. Thus, both ectopic coupling intervals and post-ectopic pauses were excluded. For frequency-domain analysis, each interval to be excluded (because of ectopic beats or artifact) was replaced by holding the previous coupling interval level throughout to the next valid coupling interval. The HRV was analyzed 2 h preoperatively, and 4 h, 12 h, and 24 h postoperatively, respectively. The following HRV indices were measured: mean RR interval (ms) and standard deviation (SD; ms); root mean square of difference of successive RRs (rMSSD; ms); proportion of adjacent RRs 50 ms different (pNN50; %); as well as low (LF; 0.04 to 0.15 Hz) and high frequency (HF; 0.15 to 0.40 Hz) power (ms2). Fast Fourier transformation was used to compute the power within the defined frequency limits for each 5-min interval. The LF/HF ratio was proposed to be an index of sympathovagal balance.

Continuous data were presented as mean ± SD. Patients’ demographic, clinical and operative characteristics were compared using Student t tests for normally distributed variables. All statistical tests were two-tailed, and a *p* < 0.05 was regarded as statistically significance.

## Results

From October 1, 2012 to June 30, 2016, a total of 463 patients consecutively underwent pulmonary lobectomy. Afterwards, 246 patients with age over sixty years old were included, and treated with VATS procedure. Based on exclusion and inclusion criteria, 112 patients undergoing video-assisted thoracic surgery (VATS) and 112 patients undergoing thoracotomy (THOR) were included in this study. The two groups were matched for age and gender.

The preoperative characteristics, including the ratio of use of β acceptor blockers for hypertension or coronary artery disease, comparable between the two groups. Altogether 39 (17.4%) patients developed AF, including 19 in VATS group and 20 in THOR group. The incidence rate of AF was comparable between the two group (16.9% *vs*17.9%, *P* = 0.79). These 39 patients developed AF at 24 h postoperatively and recovered smoothly. There were no differences in postoperative complications, including atelectasis, prolonged air leakage, pneumothorax, and pneumonitis. The mean length of hospital-stay for patients who underwent thoracotomy was significantly longer than those who underwent VATS (Table 1).

**Table 1:** Characteristics of patients undergoing lobectomy

Variables	VATS (***n*** = 112)	THORAC (***n*** = 112)	***P*** value
**Clinical data**			
Age (mean ± SD, years)	71 ± 8	71 ± 6	0.95
Male (%)	71 (63.4)	69 (61.7)	0.79
Smoking (%)	67 (59.8)	65 (58.0)	0.75
Hypertension (%)	35 (31.3)	32 (28.5)	0.78
CAD (%)	18 (16.1)	16 (14.3)	0.81
β-blockage	16 (14.2)	15 (13.4)	0.61
Diabetes (%)	12 (10.7)	10 (8.9)	0.67
Chemotherapy (%)	16 (14.3)	13 (11.6)	0.59
Heart rate (beats/min)	70 ± 12	72 ± 13	0.89
Pulmonary function			
FVC (%)	90 ± 27	97 ± 22	0.56
FEV_1_ (%)	86 ± 22	85 ± 20	0.63
Surgical procedure			
Right lobectomy (%)	47 (42)	45 (40.2)	0.82
Left lobectomy (%)	65 (58)	67 (59.8)	0.72
Pathology (%)			
Adenocarcinoma	52 (46.4)	48 (42.8)	0.58
Squamous carcinoma	49 (43.7)	52 (46.4)	0.42
Small cell lung carcinoma	6 (5.4)	5 (4.5)	0.32
Large cell lung carcinoma	5 (4.5)	7 (6.3)	0.28
Postoperative complication			
Atrial fibrillation (%)	19 (16.9)	20 (17.9)	0.79
Pneumonia (%)	2 (1.7)	3 (2.6)	0.73
Atelectasis (%)	6 (5.2)	8 (7.1)	0.61
Air leak duration (%)	14 (12.5)	12 (12.7)	0.71
Length of stay in hospital (days)	7.5 ± 3.2	13.6 ± 4.1	< 0.005

The postoperative indices of HRV were elevated significantly than preoperatively, indicating that the excitability of autonomic nerve system was increased significantly. The parameters of HRV at four-time intervals showed no difference between the two groups, including standard deviation of NN intervals (SDNN) reflecting the activity of autonomic nerves system, HF reflecting vagal activity, LF reflecting both sympathetic, and LF/HF ratio reflecting the balance between sympathetic and vagal activities (Table 2 and Table 3).

**Table 2:** Time-domain parameters of HRV

	VATS(***n*** = 112)	THOR (***n*** = 112)	***P*** value
**RR interval (ms)**			
**Pre-op 2 h**	620 ± 108	612 ± 130	0.43
**Pre-op 4 h**	683 ± 109	708 ± 104	0.89
**Post-op12h**	670 ± 118	706 ± 129	0.59
**Post-op24h**	657 ± 108	648 ± 132	0.79
**SDNN (ms)**			
**Pre-op 2 h**	35 ± 26	37 ± 28	0.81
**Pre-op 4 h**	50 ± 37	48 ± 37	0.12
**Post-op12h**	61 ± 43	55 ± 31	0.51
**Post-op24h**	63 ± 42	58 ± 33	0.77
**rMssD (ms)**			
**Pre-op 2 h**	31 ± 26	33 ± 36	0.79
**Pre-op 4 h**	42 ± 60	38 ± 58	0.08
**Post-op12h**	43 ± 41	36 ± 53	0.81
**Post-op24h**	51 ± 39	45 ± 56	0.76
**pNN50%**			
**Pre-op 2 h**	2.1 ± 4.8	2.3 ± 5.5	0.66
**Pre-op 4 h**	3.8 ± 3.1	3.2 ± 5.6	0.73
**Post-op12h**	3.5 ± 5.2	2.6 ± 5.7	0.41
**Post-op24h**	3.9 ± 5.9	2.7 ± 6.6	0.32

**Table 3:** Frequency-domain parameters of HRV

	VATS	THOR	***P*** value
**LnLF (ms**^**2**^**)**			
**Pre-op 2 h**	4.7 ± 1.2	4.8 ± 1.3	0.91
**Pre-op 4 h**	6.1 ± 1.5	5.7 ± 1.4	0.73
**Post-op12h**	5.9 ± 1.9	5.2 ± 1.5	0.59
**Post-op24h**	5.4 ± 1.9	4.9 ± 1.3	0.33
**LnHF (ms**^**2**^**)**			
**Pre-op 2 h**	4.2 ± 1.6	4.1 ± 1.5	0.88
**Pre-op 4 h**	5.8 ± 2.9	5.6 ± 1.9	0.79
**Post-op12h**	5.8 ± 2.1	5.3 ± 1.7	0.69
**Post-op24h**	4.8 ± 1.6	5.5 ± 1.8	0.61
**LF/HF ratio**			
**Pre-op 2 h**	1.6 ± 0.7	1.3 ± 1.7	0.39
**Pre-op 4 h**	2.0 ± 1.2	2.2 ± 3.1	0.67
**Post-op12h**	3.6 ± 2.0	3.0 ± 7.5	0.36
**Post-op24h**	2.4 ± 0.7	2.0 ± 2.8	0.64

## Discussion

The VAST has been widely used because of minimal invasiveness and rapid recovery. However, it’s unclear if the incidence of AF could be decreased by VAST. In a retrospective study, we found that the postoperatively incidence of AF in non-cardiac thoracic surgery was 20% [6]. The preferred surgical approach was thoracotomy. In contrast, Jaklitsch et al. reported the incidence of AF as 3.1% in 32 elderly patients undergoing VATS lobectomy [7], whereas Gharagozloo et al. reported an arrhythmia rate of 9.4% (17/179) [8]. McKenna et al. reported the largest single institution series of 1100 VATS lobectomies with a postoperative AF rate of 2.9% [9]. These data suggest that minimally invasive VATS partially by obviating surgical stress induced by rib-spreading thoracotomy, may result in a decreased incidence of AF. The difference in incidence of AF may be explained by differences in AF definition, monitoring techniques, and prevention strategies. Amar et al. observed that elderly patients undergoing a lobectomy had an incidence of AF as 27% when telemetry was used [10]. In our study, we used continuous Holter to monitor hearts for 96 h. The overall incidence of AF was 17.4%, which might be actually underestimated because of being unable to detect asymptomatic or “silent” episode that required no intervention.

Park et al. reported equal frequency of AF after lobectomy, regardless of surgical approach [11]. Falcoz et al. compared the outcome following thoracoscopic versus open lobectomy in case-matched groups of patients from the European Society of Thoracic Surgeon (ESTS) database. They observed no difference in the incidence of postoperative AF between the two groups (5.1% vs. 4.3%, *P* = 0.1433) [4]. In our present study, Holter was used to monitor the hearts of 224 patients aged over 60 years who were treated with lobectomy through VATS or thoracic surgery. All procedures were performed by the same surgeon. The pulmonary vascular and bronchus were managed using auto suture GIA to avoid the tension caused by the ligation of pulmonary vein in the open thoracic surgery. We monitored the hearts for 72 h post-operation and identified an overall incidence of AF as 17.4% (39/224). The AF occurred in 24 h after operation, and there was no difference between the two groups (19/112 vs. 20/112), consistent with previous series reports [3–5].

The etiology of AF after non-cardia thoracic surgery is poorly understood. Autonomic nerve system may play an important role [5, 12]. In our previous study, we found that the vagal resurgence in a background of increasing sympathetic activity immediately before AF onset is an important trigger factor. Amar et al. reported that the competing autonomic activity preceded the onset of postoperative AF after major thoracic surgery [5].

Could the thoracoscopic surgery offset the imbalance of autonomic nerve system that promotes the development of AF? We compared the indices of HRV between the two groups at four time points including 2 h pre-operation, 8 h, 12 h and 24 h post-operation. The postoperative indices of HRV were increased significantly compared to pre-operative ones. However, there was no difference between the two groups, indicating that the autonomic nerve system was activated in both groups. The VATS technique might not affect competition between the parasympathetic and sympathetic nerve systems.

The heart is richly innervated by the autonomic nerves [13]. Their ganglion cells are located either outside (extrinsic) or inside (intrinsic) the heart. Both extrinsic and intrinsic nervous systems are important for cardiac functions and arrhythmogenesis. The vagal nerves include axons that stem from various nuclei in the medulla. The extrinsic sympathetic nerves stem from the paravertebral ganglia, including the superior cervical ganglion, middle cervical ganglion, the cervicothoracic (stellate) ganglion and the thoracic ganglia. The intrinsic cardiac nerves are located mostly in the atria, and intimately involved in atrial arrhythmogenesis. The nerve densities were the highest in the LA within 5 mm from the PV–LA junction [14]. They were higher in the superior aspect of the left superior PV, anterosuperior aspect of the right superior PV, and inferior aspects of both inferior PVs than diametrically opposite. They were higher in the epicardial than the endocardial half of the tissue. They were not found in areas of discrete adrenergic or cholinergic predominance. Thus, adrenergic and cholinergic nerves are highly co-located not only at tissue but also at cellular levels [15, 16]. During the procedure of anatomically pulmonary lobectomy and dissection of mediastinal node, the autonomic nerves passing through pulmonary hilar and located at the pulmonary veins or surrounding the heart will be inevitably damaged, leading to unilateral denervation of cardium. This process subsequently results in imbalance in modulation of autonomic nerves in the background of increased sympathetic activity after operation. The procedures of dissection pulmonary hilus vs. systemic mediastinal nodes are similar between VATS and thoracotomy. The denervation of autonomic nerves to the heart and the influence of HRV will be comparable between the two groups, and thus resulting in similar incidence of AF.

Altogether, in our study, compare to THOR, VATS technique does not reduce the incidence of AF. The HRV was similar between the two procedures. Our findings support the role of autonomic nerves in the pathogenesis of AF after noncardiac thoracic surgery.

## Conclusion

VATS technique did not reduce the incidence of atrial fibrillation after lobectomy comparing with thoracotomy, and the change of heart rate variability show no difference between these two approaches, and these findings may support the role of autonomic nerves in the pathogenesis of AF after noncardiac thoracic surgery.

## Data Availability

The datasets used and/or analyzed during the current study are available from the corresponding author on reasonable request.

## Abbreviations

AF: Atrial Fibrillation
VATS: Video-Assisted Thoracic Surgery
THORAC: Thoracotomy
HRV: Heart Rate Variability
PVs: Pulmonary Veins
LA: Left Atrium
